# Analyzing Trio-Anthropometric Predictors of Hypertension: Determining the Susceptibility of Blood Pressure to Sexual Dimorphism in Body Stature

**DOI:** 10.1155/2021/5129302

**Published:** 2021-01-19

**Authors:** Ezemagu U. Kenneth, Uzomba Godwin Chinedu, Agbii Okechukwu Christian, P. O. Ezeonu, S. G. Obaje

**Affiliations:** Department of Anatomy, Faculty of Basic Medical Sciences, College of Medical Sciences, Federal University Ndufu Alike, Abakaliki, Ebonyi State, Nigeria

## Abstract

**Background:**

Several studies had suggested that complex body stature could be a risk factor of hypertension.

**Objectives:**

We aim to correlate body mass index (BMI), waist-hip ratio (WHR), and waist-height ratio (WHtR) of rural dwellers in Afikpo community, Ebonyi State, Nigeria, with blood pressure parameters. Furthermore, we aim to ascertain how each of the anthropometric variables affects blood pressure in men and women, respectively.

**Materials and Methods:**

A sample of 400 (200 males and 200 females) adults aged 18–89 years were selected for the correlation cross-sectional study. Data for weight, height, waist, and hip circumferences were collected by means of anthropometric measurement protocol with the aid of a calibrated flexible tape and health scale and mercury sphygmomanometer for measurement of blood pressure. A participant was classified as being hypertensive if systolic blood pressure (SBP) was >140 mmHg and diastolic blood pressure (DBP) >90 mmHg. Pulse pressure was recorded as the numeric difference of SBP and DBP.

**Results:**

The result revealed that male BMI and WHR were higher than those of females while female WHtR was higher than that of males (*P* < 0.01). The prevalence of hypertension failed to correlate with sex among participants in the study (*χ*^2^ = 0.567; *P* < 0.05). Variation in SBP and DBP of both sexes was dependent on BMI, WHtR, and waist and hip circumference, but not on WHR. The SBP of both sexes and female pulse pressure did correlate with age (*P* < 0.001). Waist circumference, BMI, and WHtR correctly predicted the variations in SBP, DBP, and pulse pressure.

**Conclusion:**

The strength of association of BMI, WHtR, and waist girth with SBP and DBP of both sexes was robust and similar, but inconsistent with WHR. Thus, a simple estimation of the trio-anthropometric predictors could serve as a means for routine check or preliminary diagnosis of a patient with hypertension.

## 1. Introduction

The skirmish with hypertension and third world health challenges has been one of the foremost global concerns. The symptomatic management of high blood pressure in most developing nations without clinical knowledge has worsened the identification of its causes thereby increasing the prevalence of hypertension especially, in sub-Saharan region of Africa. Its complications constitute approximately 25% of emergency medical admissions in urban hospitals in Nigeria [1–4]. Pulse pressure can independently predict morbidity and mortality more than other blood pressure parameters, especially, among hemodialysis and cardiovascular disease patients [5, 6]. The prevalence of hypertension among adults of age 25 is 46% in Africa, and it is the highest compared with other continents [7]. A review in Australia, with wider coverage (1968–2015), found the overall crude prevalence of hypertension to range from 2.1 to 47.2% in adults and from 0.1 to 17.5% in children [8].

Previous studies have investigated extensively the systematic reviews of the various prevalence studies of hypertension in rural and urban Nigeria [6, 9]. These studies also associated hypertension with the upgrade of lifestyles which include the type of food consumed and environment. Moreover, their findings suggested that the burdens of hypertension may overblow if not checked [10, 11]. Previous studies attempted to isolate one of the blood pressure parameters which could predict adverse cardiovascular outcomes in various patient populations. However, there is a paucity of research that considered the contributions of sexual dimorphism in stature to hypertension and pulse pressure.

Given the importance and role of anthropometric features in predicting and controlling hypertension in various Nigerian populations [6, 9], it is apposite to have a different outlook on their application in a relatively stable and ethnically homogeneous rural settlement in Nigeria. Afikpo is a centre of ancient Ibo tradition and the rural dwellers are predominantly farmers. They consume stable agricultural products and locally made dry gin as a beverage, thus, establishing a uniform lifestyle, ethnicity, and environment in the study population. Therefore, this study aimed at determining the relevance of body mass index (BMI), waist and hip girths and ratio (WHR), and waist-height ratio (WHtR) to hypertension among adult rural dwellers of Afikpo community, Ebonyi State, Nigeria. Furthermore, it aims to ascertain how each of the anthropometric variables affects systolic and diastolic blood pressure and pulse pressure in men and women, respectively.

## 2. Materials and Methods

### 2.1. Participants

A sample of 400 subjects (50% male: 50% female) were selected for the study. A convenience sampling technique was adopted specifically for the purpose of the quantitative study which gives a 5% margin of error and 95% level of confidence and accounts for subjects that are more readily accessible in the population. Subjects involved in this cross-sectional design were rural dwellers of the Afikpo community, Ebonyi State, Nigeria (age range: 18–89 years). A limited number of rural dwellers in our locality seek medical checkups. Therefore, those who showed no signs of ill-health and go about their routine activities were adjudged to be healthy. A town crier announced the aims and objectives of the study to the rural dwellers using our local dialect and assembled them at the village square for blood pressure and anthropometric data collection. The study was considered and approved by the ethics committee of the Alex Ekwueme Federal University Ndufu Alike. Only those who gave informed verbal consent and who showed neither signs of ill-health nor use of hypertensive drugs were allowed to participate in this study.

### 2.2. Methods

Standard anthropometric measurement protocol was adopted for this study. Weight and height were measured with the aid of a health scale (model RGZ-160). The subject was made to stand still without support, with their weight evenly distributed on the health scale, looking at the Frankfurt plane, while the weight and height were recorded. Weight and height were recorded to the nearest 0.1 kg and 0.1 m, respectively [12]. The waist circumference and hip circumference of each subject were measured using a nonelastic anthropometric tape to the nearest 0.1 cm. Waist circumference was measured midway between the lowest rib and the superior border of the iliac crest at the end of the normal breath out, while hip circumference was measured at the widest circumference over the buttocks [13]. BMI was calculated as weight/height^2^ (kg/m^2^), WHR was calculated as waist girth/hip girth while WHtR was calculated as waist girth/height. The systolic blood pressure (SBP) and diastolic blood pressure (DBP) of each participant were measured using a portable digital mercury sphygmomanometer (Model-ADC Sposphyg 760 Proscope Aneroid: Frank Healthcare Co. Ltd., China). The subject sat in an upright position on a chair with the feet on the floor and arm of interest resting on a table to enable the elbow placed at the heart level. A cuff was tied round the arm slightly above the medial and lateral epicondyle, making sure the inflatable part of the cuff surround 80% of the arm on bare skin. The stethoscope was placed on the cubital fossa and the readings of both the systolic and diastolic pressure were taken. Pulse pressure was recorded as the numeric difference of SBP and DBP. The authors were actively involved during data collection together with the trained research assistants, which included some nurses of the rural health posts.

### 2.3. Data Analyses

Descriptive statistics of male and female anthropometric and blood pressure parameters of the participants were carried out, and the significance of the mean difference between men and women was assessed by two-sample *t*-test (less than 0.05 of *P* value). A chi-square test was performed to test the null hypothesis that the prevalence of hypertension does not depend on sex. Thereafter, a correlation between blood pressure and anthropometric parameters was analyzed using Pearson's Product Moment Correlation. Since the strength of association of BMI, WHtR, and waist circumference with the blood pressure parameters of both sexes was robust and similar as shown in Table 1, we adopted multiple linear regression analyses. The trio-anthropometric parameters were used as factors to predict the variations in SBP, DBP, and pulse pressure of both sexes. The results were presented in Tables [1–5]. Data analyses were done with the help of SPSS version 23.0 (SPSS Inc. Chicago, IL).

## 3. Results

The statistical analysis of this study is shown in Tables 1–5.

Table 2 shows that male BMI and WHR are higher than those of females while female WHtR is higher than that of males (*P* < 0.05). The mean difference of SBP, DBP, and pulse pressure in both sexes was not significant (*P* < 0.05).

Table 3 shows that prevalence of hypertension was not dependent on sex (*χ*^2^ = 0.567; *P* < 0.05).

Table 3 shows that variation in SBP and DBP of both sexes was dependent on BMI, WHtR, waist, and hip girths. Likewise, female pulse pressure was dependent on BMI, waist, and hip girths but that of males failed to correlate with the trio-anthropometric parameters. SBP of both sexes and female pulse pressure did correlate with age (*P* < 0.001).

SBP and DBP of both sexes are predictable with the following equations:

Male prediction equations:
    SBP = 150.399–0.261 (BMI)–0.558 (waist girth)–0.018 (WHtR).
    DBP = 68.946 + 0.247 (BMI) + 0.184 (waist girth)–0.031 (WHtR).
Female prediction equations:
    SBP = 130.791–0.163 (BMI)–0.495 (Waist girth) + 0.823 (WHtR).
    DBP = 76.714 + 0.131 (BMI)–0.519 (Waist girth) + 0.840 (WHtR).

The regression analysis (Table 5) shows that regression coefficients of the predictors are significant, meaning that predictions made on the resultant regression equation are statistically robust and reliable. The regression equations are as follows: male pulse pressure = 68.639–0.876(BMI)–0.349(WG) + 0.757(WHtR) and female pulse pressure = 15.513 + 0.958(BMI) + 1.728(WG) −2.409 (WHtR). It also shows that the trio-anthropometric parameters could predict 49% and 74% pulse pressure of male and female participants, respectively.

## 4. Discussion

Hypertension (high arterial blood pressure) in cardiovascular disorders is an independent predictor of mortality. Indeed, it is often not detected on time and thereby described as a “silent killer” [14, 15]. The available means of routine check and asymptomatic nature of most cases of hypertension in sub-Sahara region of Africa could be responsible for the unprecedented prevalence of its associated mortality. The study adopted a correlation base analysis to quantify the relevance of simple and handy anthropometric parameters in determining pulse pressure and hypertension among ethnically homogeneous settlers of the Afikpo community in Nigeria. The result of the study did attempt to address the above scarcity and assumed sexual bias in the prevalence of hypertension. Although male BMI and WHR are higher than those of females while female WHtR is higher than that of male, the mean difference of SBP, DBP, and pulse pressure in both sexes was not significant (*P* < 0.05).

Notably, the incidence of male or female hypertension was 26% or 22%, respectively, using a blood pressure benchmark of 140/90 mmHg. Although the difference was not statistically significant, it was lower when compared with the results of several studies [9, 14, 16]. The reason for this could be traced to racial and environmental factors, which were controlled in this study. The SBP of both sexes and female pulse pressure correlated with age. Surprisingly, variation in male pulse pressure could not correlate significantly with any of the trio-anthropometric parameters, while that of female and SBP and DBP of both sexes did correlate with BMI, WG, and HG. Therefore, advancement in age or an appreciable increment in BMI, WG, or HG may lead to increased SBP of both sexes and pulse pressure in females.

Ultimately, elevation in SBP alone leads to an increase in the value of pulse pressure, and the amount of pressure in the arteries when the heart contracts. With aging, there is a reduction in the quantity and elasticity of elastic fibres in the arterial wall leading to arterial stiffness and loss of arterial adjustment to changes in heart stroke volume [17–19]. Ardently, we observed that most of the participants consume locally made dry gin, which could speed up metabolism and contribute to unwanted weight gain, a risk factor for hypertension. Therefore, efforts should be made to encourage these sets of people to strive for and maintain a healthy lifestyle.

The study established a relationship between body stature and blood pressure. Similarly, the authors in [20–22] reported that overweight and obesity are strong indicators of hypertension and typically substitute markers of excess adiposity. Similar to the studies [23, 24], female BMI was higher than that of males. In contrast, BMI of males was higher than that of females and the reason was attributed to racial difference [25]. The value of male WHR was higher than that of females while that of female WHtR was higher than that of males. These findings suggest the need to formulate a model using the trio-anthropometric parameters to diagnose hypertension.

Conversely, previous authors [26–28] stated that BMI has been mostly used to assess obesity and hypertension, but waist girth, waist-hip girth ratio, and waist-height ratio can predict correctly cardiovascular risks better than BMI. In contrast to the above findings, this study outstandingly revealed that the strength of association of BMI, WHtR, and waist girth with SBP and DBP of both sexes was similar and reliable, but that of WHR was inconsistent. Furthermore, the multiple regression analyses suggested that the trio-anthropometric variables could predict the variations in blood pressure parameters of both sexes. This marked contrast might be due to the failure of the above authors to measure hip girth or to statistically correlate BMI, waist-hip girth ratio, and waist-height ratio with blood pressure parameters and establish their interactions as summarized in the prediction equations which should serve as a measure to draw such conclusions. It could also be as a result of the fact that we considered a relatively stable and ethnically homogeneous rural settlement, thereby the influence of racial and environmental differences was controlled.

Furthermore, the study established a prediction equation for male and female pulse pressure, using the trio-anthropometric parameters. The trio-anthropometric parameters could predict 49% and 74% pulse pressure of the male and female participants, respectively. It implied that the variation in male and female pulse pressure might depend on the interactions of BMI, WG, and WHtR. This may be largely due to susceptibility of blood pressure variables; SBP and female pulse pressure to age and body stature and by extension justifies the consideration of a gender factor in the regression analysis.

### 4.1. Strength and Limitation of the Study

We ensured that the research assistants recruited in this study were trained adequately, before embarking on the study and also anthropometric data were sourced by repeated measurements using a standard anthropometric protocol. The sample size adopted for the population cross-sectional study was setback due to the withdrawal and noncompliance of some participants, especially, females at some stages of the study, because the available facility provided limited privacy. Furthermore, a limited number of rural dwellers in our locality seek medical checkups. Therefore, those who showed no signs of ill-health and go about their routine activities were adjudged to be healthy. Although the study did not consider the dietary intake and physical activities of each of the participants, it excluded the impact of the upgrade of lifestyles, ethnicity, and environment, since they are predominantly farmers and consume locally made dry gin as a beverage and stable agricultural products. These factors no doubt are indicators for the incident of hypertension in previous studies [29, 30]. Again, the inclusion of BMI, WHR, and WHtR in the statistical model was justified since the three parameters are widely used measures of adiposity.

## 5. Conclusion

The study provided a clear understanding of how appreciable increment in BMI, WG, and WHtR could lead to hypertension and variation in SBP, DBP, and pulse pressure, using correlation base analysis. Precisely, it revealed that an appreciable increase in female waist and hip girth with the advancement in age could increase pulse pressure and lead to hypertension. Furthermore, it suggested that SBP, DBP, and pulse pressure of both sexes were similar to each other and not mutually exclusive.

### 5.1. Recommendation

A simple estimation of the trio-anthropometric parameters could serve as a preliminary diagnosis of a patient with hypertension. However, further study is urgently needed to understand how other indicators of hypertension interact with body stature and possibly facilitate a model of intervention. It will add to available means of a routine check of hypertension and reduce significantly the prevalence of its associated mortality.

## Figures and Tables

**Table 1:** Pearson correlation coefficients of the anthropometric and blood pressure parameters of male and female rural dwellers of Afikpo community, Ebonyi State, Nigeria.

Variables	Male			Female		
	SBP	DBP	Pulse pressure	SBP	DBP	Pulse pressure
Age (years)	0.318 ^*∗∗*^	0.114	0.101	0.261 ^*∗∗*^	0.085	0.220 ^*∗∗*^
Height (m)	−0.230 ^*∗∗*^	−0.054	0.182 ^*∗∗*^	0.029	−0.048	0.071
Weight (kg)	0.228 ^*∗∗*^	0.351 ^*∗∗*^	−0.061	0.347 ^*∗∗*^	0.130	0.279 ^*∗∗*^
BMI (kg/m^2^)	0.355 ^*∗∗*^	0.386 ^*∗∗*^	0.034	0.325 ^*∗∗*^	0.147 ^*∗*^	0.240 ^*∗∗*^
Waist girth (inch)	0.154 ^*∗*^	0.245 ^*∗∗*^	−0.048	0.326 ^*∗∗*^	0.239 ^*∗∗*^	0.173 ^*∗*^
Hip girth (inch)	0.178 ^*∗*^	0.172 ^*∗*^	0.035	0.268 ^*∗∗*^	0.140 ^*∗*^	0.184 ^*∗*^
WHR	−0.025	0.110	−0.114	0.006	0.069	−0.045
WHtR	0.275 ^*∗∗*^	0.274 ^*∗∗*^	0.048	0.293 ^*∗∗*^	0.246 ^*∗∗*^	0.130

WHR = waist-hip ratio, WHtR = waist-height ratio, SBP = systolic blood pressure, DBP = diastolic blood pressure. ^*∗∗*^and  ^*∗*^Correlation is significant *P* < 0.001 and *P* < 0.05(2-tailed), respectively.

**Table 2:** Descriptive statistics of male and female anthropometric and blood pressure parameters of rural dwellers of Afikpo community, Ebonyi State, Nigeria.

	Male			Female			*P* value
	Mean ± SD	Max.	Min.	Mean ± SD	Max.	Min.	
Age (years)	35.7 ± 14.1	89.0	18.0	31.1 ± 9.2	70.0	18.0	0.000
Height (m)	1.7 ± 0.1	1.8	1.40	1.2 ± 0.1	1.8	1.5	0.000
Weight (kg)	68.1 ± 10.6	102.0	40.0	67.7 ± 11.5	112.0	42.0	0.000
BMI (kg/m^2^)	25.6 ± 13.2	37.7	17.2	26.3 ± 4.4	41.6	18.7	0.000
WG (m)	0.8 ± 0.1	1.0	0.6	0.8 ± 0.2	1.1	0.6	0.899
HG (m)	0.9 ± 0.1	1.2	0.7	0.9 ± 0.1	1.5	0.8	0.443
WHR	0.9 ± 0.5	0.1	0.7	0.8 ± 0.1	0.1	0.6	0.000
WHtR	0.5 ± 0.3	0.6	0.4	0.5 ± 0.4	0.7	0.4	0.000
SBP (mmHg)	129.7 ± 16.4	185.0	85.0	127.3 ± 18.9	212.0	84.0	0.388
DBP (mmHg)	80.2 ± 13.6	130.0	51.0	79.6 ± 13.9	129.0	47.0	0.327
Pulse pressure	49.4 ± 16.7	135.0	2.0	47.6 ± 16.1	120.0	2.0	0.270

WG = waist girth, HG = hip girth, WHR = waist-hip ratio, WHtR = waist-height ratio, SBP = systolic blood pressure, and DBP = diastolic blood pressure.

**Table 3:** Prevalence of hypertension among rural dwellers of Afikpo community, Ebonyi State, Nigeria.

Sex	Hypertension (%)	Normal (%)	*χ* ^2^	*P* value
Male	26	74	0.567	0.452
Female	22	78		

Age range (18–89 years); blood pressure cutoff points (140/90).

**Table 4:** Regression analysis summary for the trio-anthropometric predictors of SBP and DBP in male and female rural dwellers of Afikpo community, Ebonyi State, Nigeria.

	Predictors	SBP				DBP			
		Regression coefficient (B)	*P* value	OR	CI of 95%	Regression coefficient (B)	*P* value	OR	CI of 95%
Male	BMI (kg/m^2^)	−0.261	0.908	0.970	0.578–1.628	0.247	0.471	1.280	0.654–1.250
	WG (inch)	−0.558	0.162	0.572	0.262–1.251	0.184	0.794	1.202	0.302–1.479
	WHtR	−0.018	0.687	0.982	0.900–1.072	−0.031	0.588	0.969	0.865–1.085

Female	BMI (kg/m^2^)	0.163	0.174	1.177	0.931–1.489	0.131	0.256	1.140	0.914–1.422
	WG (inch)	−0.495	0.248	0.609	0.263–1.413	−0.519	0.224	0.595	0.258–1.373
	WHtR	0.823	0.205	0.227	0.638–1.340	0.840	0.198	0.232	0.645–1.319

WG = waist girth, WHtR = waist-height ratio, SBP = systolic blood pressure, DBP = diastolic blood pressure.

**Table 5:** Regression analysis summary for anthropometric parameters and pulse pressure in male and female rural dwellers of Afikpo community, Ebonyi State, Nigeria.

	Prediction % pulse pressure			
Pulse pressure (*R*^2^)	Equation 1 (male)		Equation 2 (female)	
	0.049(0.000)		0.074(0.002)	
Coefficient	
		BMI	WG	WHtR
Pulse pressure	Male r(*p*)	−0.876(0.002)	−0.349(0.393)	0.757(0.000)
	Female r(*p*)	0.958(0.003)	1.728(0.062)	−2.409(0.111)

WG = waist girth, WHtR = waist-height ratio.

## Data Availability

The database analyzed for this research includes information on all rural dwellers of male and female Afikpo people. The data can be obtained by request from your archives.

## Supplementary Materials

Supplementary Materials The correlation result of age grouping and hypertension variables of male and female rural dwellers of Afikpo community, Ebonyi State, Nigeria, is included.
