# Supplementary material for: Analyzing Trio-Anthropometric Predictors of Hypertension: Determining the Susceptibility of Blood Pressure to Sexual Dimorphism in Body Stature
Source: Int J Hypertens. 2021 Jan 19;2021:5129302. doi: 10.1155/2021/5129302 (PMC7837789; doi:10.1155/2021/5129302)
Supplement: Supplementary Materials — The correlation result of age grouping and hypertension variables of male and female rural dwellers of Afikpo community, Ebonyi State, Nigeria, is included. [file 5129302.f1.docx]

**Correlation Result of Age Grouping and Hypertension variables** **of Male and Female Rural Dwellers of Afikpo Community, Ebonyi State, Nigeria**

| Age Groups | Male | | | Female | | |
| --- | --- | --- | --- | --- | --- | --- |
|  | SBP | DBP | Pulse Pressure | SBP | DBP | Pulse Pressure |
| 30 and Less (years) | 0.185 | -0.113 | 0.274** | 0.085 | -0.116 | 0.184 |
| 31-40 (years) | 0.003 | 0.004 | -0.001 | 0.063 | 0.162 | 0.075 |
| 41-50 (years) | 0.061 | -0.070 | 0.167 | -0.070 | -0.253 | 0.120 |
| 51-60 (years) | 0.143 | -0.294 | 0.388 | -0.040 | -0.109 | 0.016 |
| 61 and above (years) | 0.377 | -0.263 | 0.528* | -0.648 | -0.463 | 0.076 |

The above table shows that pulse pressure is strongly associated with male age groups of 30 and less (p<0.001) and 61 and above (years) (p<0.05). While hypertension variables of SBP and DBP show no significant association with male and female age groups (p>0.05)
